# Estimates of CO_2 _from fires in the United States: implications for carbon management

**DOI:** 10.1186/1750-0680-2-10

**Published:** 2007-11-01

**Authors:** Christine Wiedinmyer, Jason C Neff

**Affiliations:** 1 Atmospheric Chemistry Division/The Institute for Integrative and Multidisciplinary Earth Studies, National Center for Atmospheric Research, Boulder, CO, USA; 2 Geological Sciences Department and Environmental Studies Program, University of Colorado, Boulder, CO, USA

## Abstract

**Background:**

Fires emit significant amounts of CO_2 _to the atmosphere. These emissions, however, are highly variable in both space and time. Additionally, CO_2 _emissions estimates from fires are very uncertain. The combination of high spatial and temporal variability and substantial uncertainty associated with fire CO_2 _emissions can be problematic to efforts to develop remote sensing, monitoring, and inverse modeling techniques to quantify carbon fluxes at the continental scale. Policy and carbon management decisions based on atmospheric sampling/modeling techniques must account for the impact of fire CO_2 _emissions; a task that may prove very difficult for the foreseeable future. This paper addresses the variability of CO_2 _emissions from fires across the US, how these emissions compare to anthropogenic emissions of CO_2 _and Net Primary Productivity, and the potential implications for monitoring programs and policy development.

**Results:**

Average annual CO_2 _emissions from fires in the lower 48 (LOWER48) states from 2002–2006 are estimated to be 213 (± 50 std. dev.) Tg CO_2 _yr^-1 ^and 80 (± 89 std. dev.) Tg CO_2 _yr^-1 ^in Alaska. These estimates have significant interannual and spatial variability. Needleleaf forests in the Southeastern US and the Western US are the dominant source regions for US fire CO_2 _emissions. Very high emission years typically coincide with droughts, and climatic variability is a major driver of the high interannual and spatial variation in fire emissions. The amount of CO_2 _emitted from fires in the US is equivalent to 4–6% of anthropogenic emissions at the continental scale and, at the state-level, fire emissions of CO_2 _can, in some cases, exceed annual emissions of CO_2 _from fossil fuel usage.

**Conclusion:**

The CO_2 _released from fires, overall, is a small fraction of the estimated average annual Net Primary Productivity and, unlike fossil fuel CO_2 _emissions, the pulsed emissions of CO_2 _during fires are partially counterbalanced by uptake of CO_2 _by regrowing vegetation in the decades following fire. Changes in fire severity and frequency can, however, lead to net changes in atmospheric CO_2 _and the short-term impacts of fire emissions on monitoring, modeling, and carbon management policy are substantial.

## Background

Fires cover 3–4 million km^2 ^of the globe each year and are responsible for the release of 2–3 Pg of carbon to the atmosphere [1,2]. In the Western US, the wildfires that sweep through forests during the summer months are often large, severe, and difficult to contain. A changing climate and a century of policies that encourage fire suppression, has increased the recent extent and frequency of Western US fires [3]. There are numerous well-documented effects of fire on atmospheric chemistry, pollutants, and ecosystems (e.g., [4-6]). Fire emissions impact climate through the direct emission of greenhouse gases, such as CO_2 _and methane [7] and via secondary processes, for example, by altering aerosol and ozone concentrations [8]. The impacts of fire on CO_2 _emissions to the atmosphere can be large at both the regional [9] and global [2] scales, but there is significant uncertainty regarding the magnitude, timing, and variability in CO_2 _emissions from fires. Additionally, fires result in both biological and physical changes to the land surface that affects carbon exchange in subsequent years [6] and alter surface radiative balance for several decades [10].

At both national and international levels, there is an increasing focus on the establishment of emission inventories and regulation of regional C emissions to the atmosphere. In the United States, which has to date avoided federal binding commitments to CO_2 _regulation, there is increasing activity at state and regional levels to control C fluxes to the atmosphere. One component of the emerging focus on C management is the development of international, national, and regional carbon inventory and monitoring programs. To the degree that monitoring or inventory programs focus solely on industrial activities, fires would have little impact on these activities. However, atmosphere-based regional emission monitoring efforts are strongly impacted by biosphere-atmosphere C fluxes and new monitoring and modeling tools (e.g., [11]) are being developed to deconvolve natural and human sources and sinks of carbon.

From the standpoint of atmospherically-based C monitoring programs, fire is problematic because fires tend to be extremely variable in both space and time, and because emission estimates from fires tend to be highly variable and uncertain (e.g., [2,12-14]). The atmosphere integrates CO_2 _emissions from many sources and so the variability and uncertainty in fire CO_2 _emissions has the potential to propagate significant uncertainty through regional C monitoring programs. An effective C management policy will require a monitoring framework that is accurate and spatially resolved. Fires complicate the implementation of these tools because the CO_2 _emitted from fires may reduce the accuracy of terrestrial sources and sink estimates from monitoring efforts.

There has been an active and ongoing discussion about the role of biosphere C exchange in CO_2 _mitigation and the Kyoto Protocol includes a limited set of biosphere-based forestry and agricultural-management options that can be used to partially offset fossil fuel emissions [15]. From a policy standpoint, the role of fire in C policy development depends on the scope of any mandated emission reductions and whether biogenic sources are incorporated into emission inventories; to date this has not been the case for 'natural' emission sources such as fire, but the role of these fluxes in future policy remains uncertain. Outside the scope of treaties or national emission policy development, terrestrial C fluxes are also playing a role in the largely unregulated C offset/sequestration industry through the use of terrestrial C sequestration techniques. The large biosphere/atmosphere C fluxes have led to extensive study of both the capacity of terrestrial ecosystems to sequester C and the potential duration of terrestrial sinks [16,17]. However, there is also growing concern regarding the tendency for the leakage of stored C from terrestrial sinks [15], as this leakage has the potential to reduce the efficiency of industrial emission reductions.

Fire is one of the largest potential risks to loss of stored terrestrial C and is a loss pathway that is difficult to quantify due to the high degree of spatial and temporal variation in fire emissions. At multi-decadal time scales, wildfires have a near neutral effect on atmospheric CO_2_: forest regrowth balances punctuated C losses due to combustion, assuming that fire return intervals remain constant [18]. However, on the shorter time scales of legislative agreements, international accords, or in the context of the emerging markets for carbon offsets, fires can lead to rapid, large emissions of C and add considerable uncertainty to projections of decadal scale ecosystem carbon budgets [6,19].

In the Western US, fires can be widespread in a state one year and virtually absent the next (e.g., [13]). In a study of emissions in Canada, wildfires contribute the equivalent of 18% of emissions from the energy sector of the country with a year to year range in emissions that varies from 2 to 75% [9]. Although fires may not become a target for national emission regulations, the fluxes from these events, if they are as significant as Amiro et al. [9] report, are clearly important short-term influences on regional C emission patterns. The combination of uncertainty in emission estimates due to the spatial heterogeneity in burns, and uncertainty regarding the degree of combustion of aboveground biomass and soil organic matter stocks [20] makes attribution of C fluxes associated with fire very challenging. In the context of C monitoring, the potential of fires to match, or even exceed, industrial fluxes in some settings and the high degree of uncertainty associated with these fluxes could make it difficult to develop regional C monitoring techniques that would be capable of providing sufficient source/sink information for policy development or implementation.

Fire return intervals in forested US ecosystems vary, but range from decades in semi-arid interior forests to centuries for coastal ecosystems [21]. There has been much debate over the role of historical land management practices, such as fire suppression, in contemporary fire and forest growth patterns and a growing discussion of how wildfires will respond to climate change (e.g., [3,22,23]). The long duration of forest regrowth between fire events and the variability in the magnitude of C emission during fire highlights the uncertainty of this aspect of terrestrial C cycling. In the Kyoto protocol, the complex nature of terrestrial sources and sinks led to a relatively narrow definition of the types of terrestrial C sequestration activities that could be used to meet treaty objectives [24]. These sequestration activities thus far have been largely constrained to agricultural management and reforestation projects, although there has been a vigorous and ongoing debate about the appropriate scope of terrestrial C sequestration activities [25]. At regional and national levels, terrestrial sinks driven by historic land use change, such as reforestation efforts, can be sizeable [26] and may represent an attractive target in future C mitigation negotiations. Similarly, fire mitigation programs such as forest thinning may reduce the severity or extent of fires, but may also have uncertain impacts on sequestered carbon (depending on the fate of C removed from forests). From this standpoint, the potential for C losses from fire represents a risk to C sequestration potential and a factor that needs to be considered in discussions regarding appropriate credit for terrestrial sinks in atmospheric C mitigation.

In this study, we evaluate the role that fire plays in carbon emissions from a number of states throughout the US. The motivation, following Amiro et al., [9], is, in part, to assess the degree to which fire can influence regional carbon budgets and the year to year and state to state variability of the potential impacts. This is the first study of which we are aware that includes the spatial and temporal resolution of fire CO_2 _emissions for the US, and assesses the importance of these emissions compared to fossil fuel burning CO_2 _emissions. We also focus on the role that fire may play in longer-term ecosystem C budgets by comparing fire emissions to Net Primary Productivity (NPP) in a range of ecosystems at a regional level. Through these comparisons, our goal is to more clearly delineate the role that fire is playing in regional C budgets with the hope of providing some insight into the impact that fire may have on both C monitoring and management plans in the future.

## Results and discussion

### CO_2 _emissions from fires

Daily CO_2 _emissions from fires in North America were estimated for 2002 through 2006 using the methods described by Wiedinmyer et. al. [13]. Annually, the average CO_2 _emitted from fires in the lower 48 (LOWER48) states from 2002–2006 is estimated to be 213 (± 50 std. dev.) Tg CO_2 _yr^-1 ^and 80 (± 89 std. dev.) Tg CO_2 _yr^-1 ^in Alaska. There is substantial variation in the overall magnitude of annual emissions from states in the US, ranging from the average of 80 Tg of CO_2 _in Alaska to < 0.01 Tg CO_2 _in Rhode Island and Vermont. Emissions from the Northeastern and Midwestern US states tend to be very small; the annual emissions from the US are dominated by the Western and Southeastern US states. For many Western and Southeastern US States, there are large annual fire emissions of CO_2 _averaging ~10 Tg CO_2 _(with an average coefficient of variance of more than 50%). The Northeastern states have the least amount of emissions per area: Vermont, Rhode Island, Maine, and New Hampshire all have an average annual fire emission of <1 metric ton CO_2 _km^-2^. The Southeastern and Western states have the largest amount of CO_2 _from fires: Alabama, Florida, Georgia, Louisiana, and Washington all have an average annual fire emission > 75 metric ton CO_2 _km^-2^.

The interannual variability in the annual emission estimates is substantial. In the LOWER48, the annual emissions from year to year vary as much as a factor of 1.8, and in Alaska, the annual CO_2 _estimates vary by over an order of magnitude. Overall, the interannual variance of fire emissions in the Southeastern US is lower than in the Western US. This interannual variability could arise from several causes, including changes in meteorology and climate (e.g., drought) and land management practices that deal with agricultural and prescribed burning.

Fires occur within the US for a number of reasons, including wildfires started from both natural and anthropogenic causes, prescribed burning, and burning for agricultural purposes. An analysis of the fire emission estimates presented here shows that the majority of the emissions from fires in the US are from needleleaf forests. For 2006, needleleaf forests are estimated to emit 78% of the CO_2 _emissions from continental US fires. This suggests that, although important, natural and prescribed burning in grasslands and burning in croplands for agricultural purposes does not contribute significantly to the overall annual US CO_2 _fire emissions inventory. CO_2 _emissions from grasslands account for 5% of the 2006 estimated fire emissions inventory, and emissions from croplands contribute <3%. In both the Western and the Southeastern US, 86% of the estimated 2006 CO_2 _emissions come from needleleaf forests.

The amount of area burned for management practices (prescribed burns) varies by region. In the Southeastern US, the majority of acreage burned is via prescribed burns. According to the National Interagency Fire Center (NIFC; [27]) less than one third of the reported area burned in 2006 in the Southeastern states was due to wildfires; two thirds of the area burned was the result of prescribed burns. In Alabama, 94% of the 2006 reported burn area was attributed to prescribed burns. Since prescribed burns in the Southeastern US tend to occur between November and April [28] and the majority of emissions in this region come from needleleaf forests, we assume that much of the emissions through the spring and fall months (discussed below) can be primarily attributed to prescribed burns in forested areas.

In the Western US, fire-related CO_2 _emissions are dominantly related to wildfire activity. A report for the Western Regional Air Partnership [29] estimates that 57% of the acreage burned in 2002 in the Western US States was due to wildfires, 23% for agricultural purposes, and the remainder for land management practices. Although the percentage of agricultural burned area was significant, the amount of biomass burned, and therefore the emissions, were relatively small in the overall inventory.

### Seasonal variation in fire CO_2 _emissions

**Figure 1:**
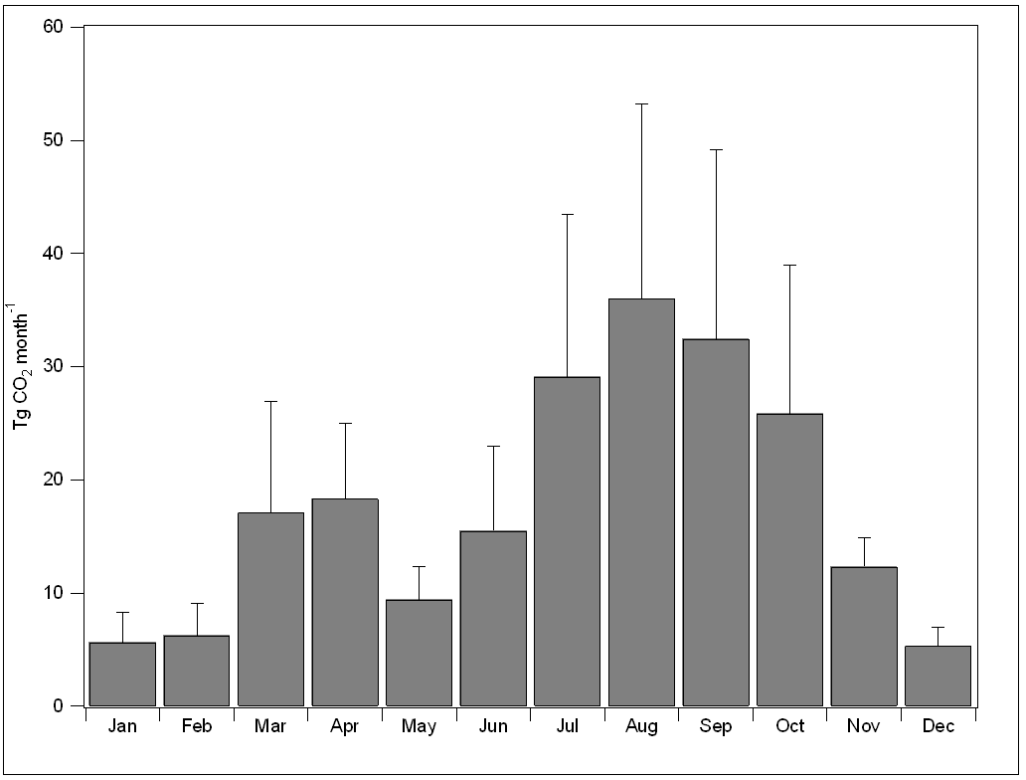
**Annual emissions of CO2 from fires**. Monthly emissions of CO_2 _from fires for the LOWER48, averaged for 2002–2006. The error bars represent the standard deviation of the monthly emissions for the 5 years.

**Figure 2:**
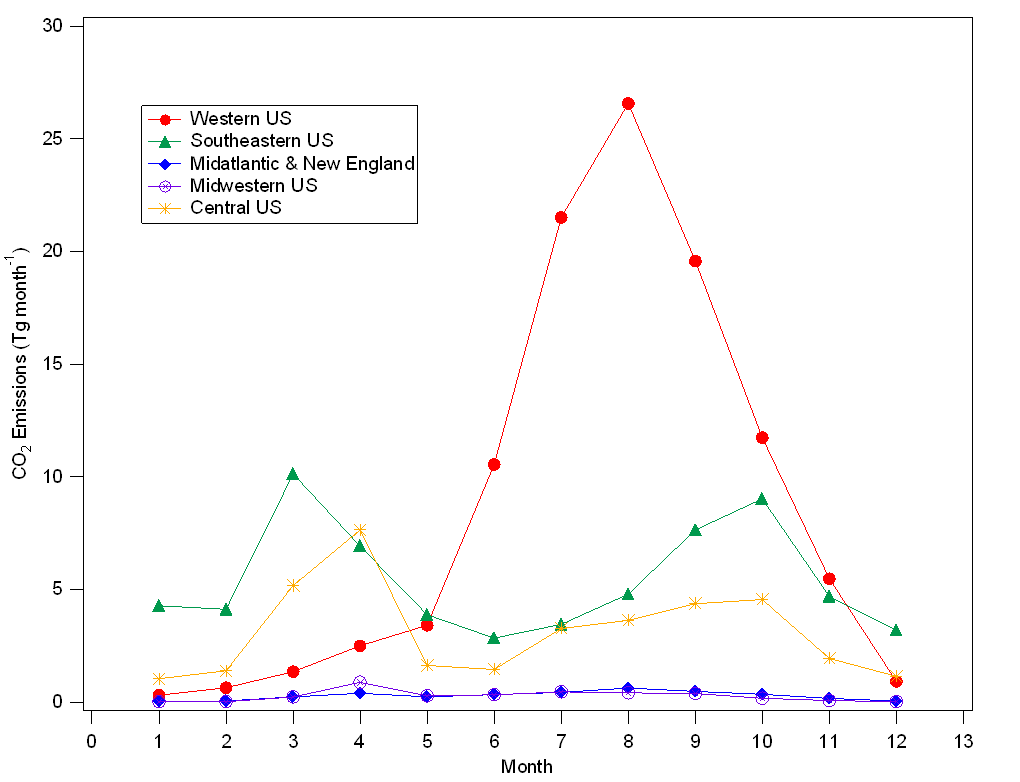
**Monthly CO_2 _emissions by region**. Annually-averaged CO_2 _emissions (2002–2006) from fires for five US regions. (Western US = AZ, CA, CO, ID, MT, NM, NV, OR, UT, WA, WY; Southeastern US = AL, FL, GA, LA, MS, NC, SC, TN; Mid-Atlantic & New England = CT, DE, MA, MD, ME, NH, NJ, NY, PA, RI, VA, VT, WV; Midwestern US = IL, IN, KY, MI, OH, WI; Central US = AR, IA, KS, MN, MO, NB, ND, OK, SD, TX)

There is strong seasonal variation in fire CO_2 _emissions, with regional differences in the peak emissions across the US. Generally, the monthly emissions of CO_2 _from fires in the LOWER48 have two peaks: a small peak during the spring months (March and April) and a larger peak during the summer months (Figure 1). These two peaks are driven by the timing of fires in two distinct portions of the US, with spring fire emissions dominated by fires in the Southeastern and Central US, and summer fire emissions driven by emissions for the Western US (Figure 2).

**Figure 3:**
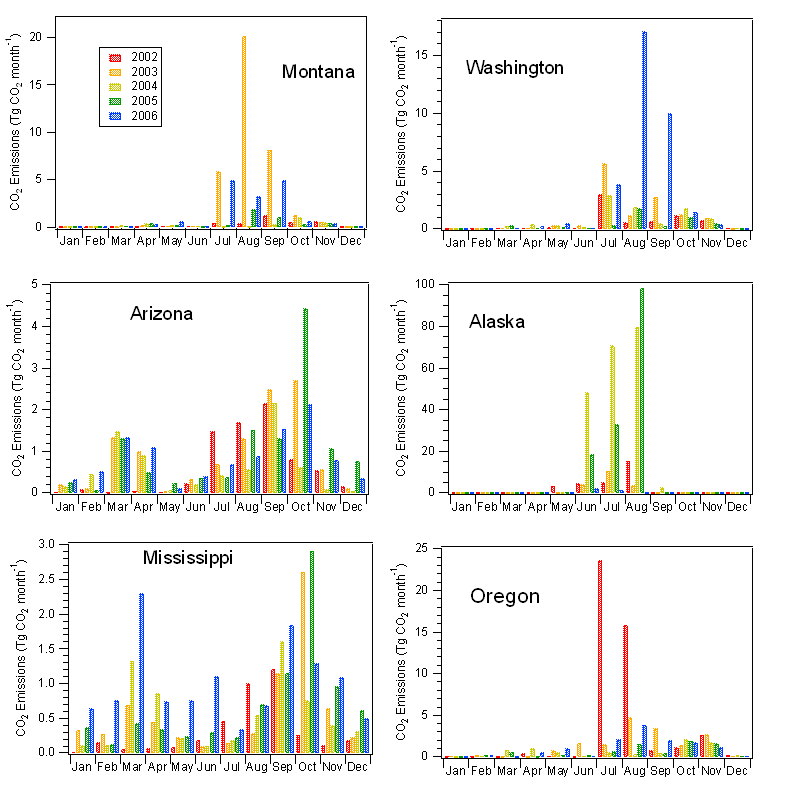
**Monthly state CO_2 _emissions**. Monthly emissions of CO_2_ from fires for   selected states.

Large, periodic fires can cause massive fluxes of CO_2 _to the atmosphere. Figure 3 shows monthly CO_2 _release from fires from six states including Alaska, four western US states, and Mississippi. These results illustrate the extreme variability in emissions associated with large fire events, such as the Columbia Complex fire in Washington in August 2006, or the Biscuit fire in Oregon in July of 2002, during which more than 15 Tg of CO_2 _was released from each of these states.

In 2002, the Biscuit fire burned in Oregon from mid-July to September. The emissions from this fire were exceptionally large and drove the peaks in CO_2 _emissions for July and August 2002 for Oregon (Figure 3). Estimates using the methods described here (see Methods Section below) predict 4.9 Tg C (from CO2) and 5.3 Tg C (from CO2 and CO) from the Biscuit fire (in Oregon only). Law et al. [30] used a simple method, based on the reported burn area and an assumed carbon loading, to estimate 4.1 Tg C from the same fire. The sizeable difference in emission estimates emphasizes the large uncertainty associated with estimating C emissions from fires. Estimates of fire emissions of CO_2 _depend on a wide range of factors, including the severity and type of burns, as well as the spatial heterogeneity of vegetation and fire intensity [2,6]. Combined, these factors make it exceptionally difficult to accurately measure C emissions from field-based techniques, regardless of methods used. Unfortunately, remote sensing-based methods also result in highly uncertain C flux estimates for fire, and there is currently no clear method available to reduce these uncertainties [19]. Given these complexities, the flux estimates for the Biscuit fire made by this study and the Law et. al. [30] study are probably about as similar as can be expected. Law et. al. [30] applied a reported burn area, while the method employed in this study applied a burn area based on remote sensing observations. Both methods used different fuel loading estimates and emission factors. The impact of inherent uncertainty in emission estimates is that the high degree of variability (e.g., >25% of the flux) in fire emission estimates is not likely to be reduced soon and has implications for both our understanding of fires in the global carbon cycle and our ability to monitor and assess the causes of biosphere-atmosphere fluxes at a regional scale.

### Fires and regional CO_2 _emissions

A striking implication of very large wildfires is that a severe fire season lasting only one or two months can release as much carbon as the annual emissions from the entire transportation or energy sector of an individual state. While the long-term atmospheric implications of wildfire and fossil-fuel C release can be strikingly different, the pulsed emission releases from wildfire events can match or even exceed monthly or annual industrial emissions on a regional basis. To examine the role of wildfire in the context of industrial emissions, we compare national and state level emissions of CO_2 _from fossil fuel combustion to our estimated fire emissions of CO_2_.

**Figure 4:**
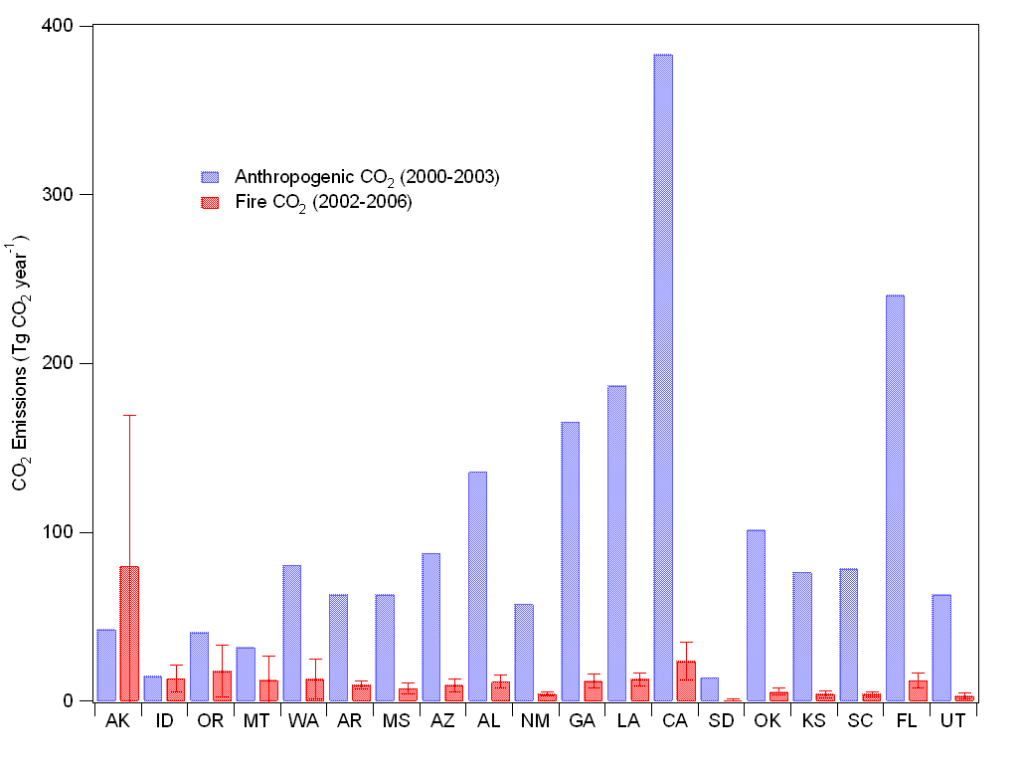
**Annual CO_2 _emissions by state**. Annually-averaged anthropogenic emissions (2000–2003) of CO_2_ and   annually-averaged CO_2_ emissions (2002–2006) from fires for states where   average fire emissions greater than 5% of the states' anthropogenic   emissions. The error bars associated with the fire emission estimates   represent the standard deviation of the annual emissions for 2002–2006.

Annually, for the continental US (not including Washington D.C.), the average CO_2 _emissions from all fossil fuel burning (FFB) sources from 2000 – 2003 were 5738 Tg CO_2 _[31]. Annual average CO_2 _emissions for 2002 – 2006 from fires in the continental US was 293 Tg CO2, corresponding to the equivalent of 5.1% of the annual FFB emissions from 2000–2003 (and 5.4% of the average from 1990–2003). Depending on the year, emissions from fires for the entire Continental US were equivalent to as little as 4% of the FFB emissions, and as much as 6%. However, tHowhis is for the entire U.S; on a state-level, the importance of fire emissions of CO_2 _relative to FFB emissions is much different. There are eight states (Alaska, Idaho, Oregon, Montana, Washington, Arkansas, Mississippi, and Arizona) where the annually-averaged (2002–2006) fire emissions are equal to more than 10% of the state-level FFB CO_2 _emissions, and eleven other states whose fire emissions equal more than 5% of the state-level CO_2 _emissions (Figure 4, Additional Table 1). In the case of Alaska, annually-averaged fire emissions of CO_2 _(2002–2006) are consistently greater than the annually averaged (2000–2003) emissions from FFB (Figure 4). For the states located in the Western and Southeastern US, average annual fire emissions of CO_2 _range from the equivalent of 2–4% of FFB emissions in North Carolina, Colorado, and Wyoming, to 89% of emissions in Idaho. (It should be noted, however, that Idaho does not have any coal-fire power plants, which emit large amounts of CO_2_). For the Western US States, fire CO_2 _emissions on average are equivalent to 11 ± 4% of annual FFB CO_2 _emissions, and for the Southeastern US fire CO_2 _emissions are equivalent to 6 ± 2% of annual FFB CO_2 _emissions.

The relative importance of CO_2 _emissions from fires to regional C emissions varies seasonally and annually. For example, during particularly intense fire years, such as 2006 in Idaho, the emissions of CO_2 _from fires in Idaho were 1.6 times higher than all of the annually-averaged (2000–2003) FFB emissions from that state, and nearly double the mean annual fire CO_2 _emissions for the state for 2002–2006. Similarly, in 2006, Montana and Washington experienced CO_2 _emissions from fires during the year that were equivalent to ~47 and 42% of the total annual state-level FFB CO_2 _emissions, respectively. In addition to significant interannual variation, regional fires are typically active for just a few months of the year. The monthly emissions of CO_2 _from fires for 2002 through 2006 for six selected states are shown in Figure 3. Alaska, Montana, Washington, and Oregon all show large summer peaks in wildfire CO_2_emissions that are of the same magnitude or greater than the CO_2 _from FFB sources during those months.

In California, the annual FFB emissions inventory of CO_2 _is the largest in the country behind Texas (362 Tg CO_2 _yr^-1 ^averaged from 1990–2003). Even so, the annual averaged emissions of CO_2 _from fires are significant (24 Tg CO2 yr^-1^; equivalent to 6% of the FFB emission estimates). Although the ratio of annual state-level CO_2 _emissions from fires to FFB sources is fairly low, and California does not have significant coal-fire power plant CO_2 _emissions, this ratio is also subject to substantial variation. By the end of October 2003, wildfires burned more than 750,000 acres, producing the equivalent of 49% of the monthly CO_2 _emitted by FFB sources for state. This occurred in more than one year that we investigated. The major wildfires in September 2006, including the Day Fire in Southern California, produced an estimated 16 Tg CO_2 _for that month, equivalent to approximately 50% of estimated total monthly FFB emissions for the entire state. Thus, even in highly industrialized regions of the country with significant FFB CO_2 _emissions, fires can contribute significant amounts of CO_2 _to the atmosphere. These fires not only impact regional CO_2 _fluxes, but can also impact visibility and air quality. Phuleria et. al. [5] shows how the emissions from the October 2003 Californian fires increased air pollutant concentrations, most notably particulate matter with diameters less than 10 μm (PM10), throughout the Los Angeles Basin.

### Multi-decadal implications of fire C release

Fires represent a potentially large short-term release of carbon that is largely offset over longer time scales (decades) by the uptake of atmospheric carbon associated with forest regrowth. From this standpoint, fires and fossil fuel emissions have entirely different effects on atmospheric CO_2 _levels with the expectation that in the absence of changes in frequency or intensity, fire emissions would be balanced over a period of several decades by forest regrowth and C assimilation. To evaluate the magnitude of C released from fire in the context of annual plant C sequestration, we compared emission estimates from fire to annual estimates of Net Primary Productivity (NPP; gC m^-2 ^year^-1^) derived from MODIS satellite observations ([31-35]) for 2000 through 2005. Annually-averaged NPP (2000–2005) by state is estimated from these base datasets (Additional File Table 1). For the LOWER48, the annually-averaged NPP was estimated as 9369 Tg CO_2 _yr^-1^. On an annual basis, fires result in a release of the equivalent of 4% of the annual NPP flux in both the Western and the Southeastern US. However, this is highly variable. For example, average annual fire emissions of CO_2 _range from 0.7–1.4% of estimated NPP in North Carolina, Colorado, and Wyoming, to more than 6% for Arizona, Idaho, and Louisiana. For the Western US, fires on average represent 3.8 ± 1.5% of annual average NPP, similar to the results for the Southeastern US, where CO_2 _from fires is 3.6 ± 1.1% of annual average NPP.

The large conversion of terrestrial biomass to CO_2 _during a fire is largely balanced over longer time scales by the uptake of C in regrowing forest. In North American Boreal ecosystems, there commonly is a period of several years to a decade during which C is lost from ecosystems, followed by several decades to a century of C uptake in regrowing forests [6,36]. However, fire regimes and intensity are changing for at least some portions of the US [3,21], and following European settlement of the Western US, the fire frequency in some forests was reduced [37] leading to an accumulation of C in terrestrial systems. The relatively large fraction of NPP that is currently lost to fire in a number of Western US ecosystems represents, in part, the return of some of this historically accumulated C to the atmosphere, and sets the stage for future C uptake in these forested ecosystems. The historic and future impact of fire emissions on atmospheric CO_2 _also depends on the frequency and intensity of fires in the 21^st ^century. A shortening of fire return intervals, increases in area burned, and/or increases in fire severity can lead to net emissions of CO_2_, even on a multi-decadal times scale [6,10,38]. With changing climate and projected increases in burned area in the US [39,40], there is a significant potential for additional net release of C from the forests of the United States due to changing fire dynamics in the coming decades.

## Conclusion

Fires represent a large and highly variable component of the US carbon budget. This study illustrates the high degree of spatial variability in fire CO_2 _emissions with exceptionally large fluxes of CO_2 _due to wildfire in the Western US and large emissions from controlled burns and forest management activities in the Southeastern US. In some Western US states, such as Alaska and Idaho, the annual emission of CO_2 _from wildfire in some years equals or exceeds the emissions from fossil fuel combustion. Even in states with large FFB CO_2 _sources, such as California, fires can be a significant annual and highly significant seasonal component to the regional C budget.

The long-term impacts of CO_2 _emissions from fire are considerably different than from fossil fuel burning emissions because fire emissions are at least partially balanced over decades by forest regrowth and terrestrial C sequestration. Changing climate and fire regimes, however may lead to fire emissions that increasingly diverge from historical means. Over shorter time periods fires, with their inherently uncertain emission estimates, represent a major hurdle to the establishment of accurate C source and sink accounting based on atmospheric CO_2 _observations. While isotopic and tracer techniques could certainly aid in the reduction of uncertainty in regional C inverse modeling, fires represent a level of complexity in terrestrial C dynamics that deserve increased attention.

## Methods

### Fire emission estimates

A simple modeling approach, described by Wiedinmyer et. al. [13], was used to calculate the daily fire emissions of carbon dioxide (E_CO2_) in North America from 2002 through 2006. E_CO2 _was calculated as:

E_CO2 _= A(x,t) * B(x,t) * EF_CO2_

where A(x,t) is the area burned at location x and time t, B(x,t) is the biomass burned at location x and time t, and EF_CO2 _is an emission factor, or the mass of CO_2 _that is emitted per mass of biomass burned.

With this method, fire location and timing is determined with the MODIS Active Fire product. The MODIS instruments aboard the NASA Terra and Aqua satellites each provide approximately twice-daily passes over North America. These daily fire detections were processed by the US Forest Service Remote Sensing Applications Center for 2002 through 2006 using the MODIS Active Fire data developed by the UMD Rapid Response team [41].

The fuel loading at each fire was determined using a combination of satellite products. The Global Land Cover 2000 (GLC2000) dataset is used to characterize the ecosystem type for each identified fire. The GLC2000 identifies 29 different land cover classes in North and Central America at a 1 km^2 ^resolution [42]. For each land cover class, a total fuel loading has been assigned using a combination of values found in the literature [13]. The fraction of woody and herbaceous fuels associated with each class was determined using information from the Fuels Characterization Classification System (FCCS; [43,44]). The fraction of forest, herbaceous cover, and bare ground at each fire was determined using the Vegetation Continuous Fields (VCF) MODIS product, scaled to 1 km^2 ^[45,46]. The amount of biomass burned was assumed to be a function of forest cover (where > 60% tree cover is considered forest, 40–60% tree cover is considered Woodlands, and <40% tree cover is considered Grasslands), following the methods applied by Ito and Penner [47].

For the results shown here, each detected fire was treated as an individual fire. Based on the nominal resolution of the MODIS instruments, the total possible area burned for each fire pixel was assumed to be 1 km^2^. For each fire detection, the 1 km^2 ^was scaled to the amount of bare cover assigned at that spot by the VCF product. For example, if the bare cover was 20% at a fire point, the area burned was estimated to be 0.8 km^2^. Using this methodology, daily fire emissions of CO_2 _were estimated for 2002 through 2006. Only emissions from the US are presented in this paper.

### Fire emission estimate uncertainty

The emissions of CO_2 _from fires are highly uncertain due to the combined errors and uncertainties in the model framework and inputs. Uncertainties in the fire emission estimates may arise from the satellite detections of the fires, the assumptions made in the fuel loading and amount of fuel burned, the estimated area burned, and the assigned emission factors. The Active Fire satellite product produces daily fire detections. This product is not screened for missing data, and does not flag those areas obstructed by clouds. The timing of the satellite detections and the inability to detect fire through clouds can lead to missed detections and an underestimation of fire detections [41,48]. The area burned assigned to each pixel (1 km^2^) is considered an upper estimate. The fuel loadings associated with each general land cover classification are taken from few studies, and in reality are highly variable.

Wiedinmyer et. al. [13] were unable to assign a quantitative assessment of uncertainty on the emission estimates using the described modeling technique. However, they predict that the uncertainties can be over a factor of two. When compared to other estimates of CO_2 _emissions from fires, these estimates are within this uncertainty. For the Conterminous US, the Global Fire Emissions Database, version 2 (GFEDv2, [2]) predict emissions of CO_2 _that are approximately two to five times lower than those estimates here. Other models used to predict emissions from fires are much closer to the values predicted here. A more comprehensive intercomparison of emission estimates of CO from fire emissions models for the US is described by Al-Saadi et. al. [12]. In general, the emissions from the methodology used here are higher than those predicted by the GFEDv2, but lower than those predicted by a NOAA product [12]. To consider the uncertainty associated with the emission estimates, we assign a factor of at least 2 to the estimates.

The validation of fire emission estimates is difficult, since the emissions from fire to fire are highly variable, and direct flux measurements from fires are extremely difficult. Inverse modeling of fire emissions using *in situ *measurements or satellite observations provides a means to constrain fire emission estimates: however, these methods can not provide a direct quantification of emissions from fires. The uncertainty in the fire emission estimates, along with the variability in the spatial and temporal allocation of these emissions, adds further complications for efforts to constrain C fluxes with monitoring and modeling techniques. Future work is needed not only to better quantify emissions from fires, but to better constrain the uncertainties associated with the estimates.

### Net Primary Productivity

The Net Primary Productivity (NPP) is defined as the rate at which biomass grows in an ecosystem. It is often used as a measure of carbon uptake by vegetation, or carbon stored in vegetation. For this study, the annual NPP values determined from the MODIS Satellite instruments were used [31-35]. This product provided annual NPP values (gC m^-2 ^year^-1^) with a spatial resolution of 1 km^2 ^for the continuous US Annual NPP values (TgCO_2 _yr^-1^) for each of the 6 years (2000–2005) were averaged for each state in the continuous US.

### Fossil fuel burning emissions of CO_2_

To evaluate the importance of biomass burning emissions relative to those from fossil fuel burning, the US Department of Energy report of annual CO_2 _emissions from fossil fuel combustion for the country [31] is used. The annual total CO_2 _emissions by state from 1990 to 2003, was published in April 2007 [49]. This inventory does not include all industrial sources, but is the most complete inventory of which we are aware.

## Competing interests

The author(s) declare that they have no competing interests.

## Authors' contributions

CW performed the fire emission estimates and processed the DOE FFB emissions and the NPP data. CW and JN both analyzed the results and contributed equally to the manuscript. Both authors have read and approved the final manuscript.

**Table 1:** Annual CO_2 _emissions from fires (Tg CO2 yr-1). The annual estimated CO_2 _emissions from fires(Tg yr^-1^) for the LOWER48 and for Alaska.

Year	LOWER48	Alaska
2002	193	28
2003	244	18
2004	157	201
2005	191	150
2006	283	3

**Table 2:** Annual CO_2 _emissions from fires for different US regions. The annually averaged (2002–2006) CO_2 _emissions (Tg yr-1), standard deviation, and the coefficient of variation for 5 regions of the LOWER48.

Tg CO_2 _yr^-1^			
Regions	Ave. Annual Emissions	Standard Deviation	Coefficient of Variation

Western US	105	42	40
Southeastern US	65	20	31
Central US	37	10	26
Mid-Atlantic & New England	3	1	20
Midwest	3	1	17

*Where*.

Western US = NM, CO, WY, MT, ID, UT, NV, AZ, CA, OR, WA.

SE US = LA, MS, AL, FL, GA, SC, NC, TN.

Central US = TX, OK, MO, KS, NB, SD, ND, IA, AR, MN.

Mid-Atlantic & New England = ME, VT, NH, RI, CT, MA, NY, PA,. NJ, DE, MD, WV, VA.

MidWest = WI, IN, IL, OH, KY, MI.

## Supplementary Material

Additional file 1 Emissions of CO_2 _from FFB and NPP for each state. The monthly and annual averaged CO_2 _emissions from each state are provided. Additionally, the FFB emissions and the annual estimated NPP are also given for each state. Click here for file
