# Supplementary material for: Estimates of CO2 from fires in the United States: implications for carbon management
Source: Carbon Balance Manag. 2007 Nov 1;2:10. doi: 10.1186/1750-0680-2-10 (PMC2203970; doi:10.1186/1750-0680-2-10)
Supplement: Additional file 1 — Emissions of CO2 from FFB and NPP for each state. The monthly and annual averaged CO2 emissions from each state are provided. Additionally, the FFB emissions and the annual estimated NPP are also given for each state. [file 1750-0680-2-10-S1.doc]

# Additional files

### Additional file 1 – Emissions of CO_2_ from FFB and NPP for each state.

|  |  |  |  |  |  |  |  |  |  |  |  |  | Tg CO_2_ year^-1^ | |  |  |  |
| --- | --- | --- | --- | --- | --- | --- | --- | --- | --- | --- | --- | --- | --- | --- | --- | --- | --- |
|  | Monthly Emissions from Fires (Tg CO_2_ month^-1^) | | | | | | |  |  |  |  |  | Annual Fire Emissions | | FFB Emissions | | NPP |
| State | JAN | FEB | MAR | APR | MAY | JUN | JUL | AUG | SEP | OCT | NOV | DEC | Annual average (2002-2006) | Standard Deviation | Annual Average (1990 - 2003) | Annual Average (2000-2003) | Annual Average (2000-2005) |
| Alabama | 0.5 | 0.6 | 1.8 | 1.0 | 0.4 | 0.3 | 0.3 | 0.9 | 2.0 | 2.3 | 1.1 | 0.6 | 12 | 4.0 | 127 | 136 | 216 |
| Alaska | 0.0 | 0.0 | 0.0 | 0.0 | 0.7 | 15.3 | 24.0 | 39.4 | 0.5 | 0.0 | 0.0 | 0.0 | 80 | 89.3 | 40 | 42 |  |
| Arizona | 0.1 | 0.1 | 0.1 | 0.2 | 0.5 | 4.1 | 1.8 | 0.3 | 0.6 | 0.9 | 0.5 | 0.2 | 9 | 3.8 | 75 | 88 | 152 |
| Arkansas | 0.2 | 0.2 | 1.1 | 0.7 | 0.1 | 0.3 | 0.7 | 1.2 | 1.9 | 2.1 | 0.6 | 0.3 | 9 | 2.2 | 58 | 63 | 207 |
| California | 0.1 | 0.3 | 0.4 | 0.5 | 0.5 | 1.2 | 3.6 | 4.0 | 6.5 | 4.9 | 1.1 | 0.4 | 24 | 11.1 | 362 | 383 | 475 |
| Colorado | 0.0 | 0.0 | 0.1 | 0.2 | 0.2 | 1.2 | 0.6 | 0.6 | 0.2 | 0.1 | 0.0 | 0.0 | 3 | 4.1 | 77 | 90 | 245 |
| Connecticut | 0.0 | 0.0 | 0.0 | 0.0 | 0.0 | 0.0 | 0.0 | 0.0 | 0.0 | 0.0 | 0.0 | 0.0 | 0 | 0.0 | 41 | 42 | 22 |
| Delaware | 0.0 | 0.0 | 0.0 | 0.0 | 0.0 | 0.0 | 0.0 | 0.0 | 0.0 | 0.0 | 0.0 | 0.0 | 0 | 0.0 | 18 | 17 | 8 |
| Florida | 1.6 | 1.7 | 2.0 | 1.5 | 1.1 | 0.6 | 0.7 | 0.6 | 0.6 | 0.6 | 0.7 | 0.7 | 12 | 4.5 | 216 | 241 | 320 |
| Georgia | 0.8 | 0.7 | 2.7 | 1.9 | 0.8 | 0.3 | 0.5 | 0.5 | 0.9 | 1.3 | 0.8 | 0.6 | 12 | 4.1 | 152 | 166 | 282 |
| Idaho | 0.0 | 0.0 | 0.1 | 0.3 | 0.4 | 0.1 | 1.0 | 5.1 | 4.2 | 1.7 | 0.5 | 0.0 | 13 | 8.1 | 14 | 15 | 206 |
| Illinois | 0.0 | 0.0 | 0.1 | 0.2 | 0.0 | 0.1 | 0.1 | 0.1 | 0.1 | 0.0 | 0.0 | 0.0 | 1 | 0.1 | 215 | 230 | 176 |
| Indiana | 0.0 | 0.0 | 0.0 | 0.1 | 0.0 | 0.0 | 0.0 | 0.0 | 0.0 | 0.0 | 0.0 | 0.0 | 0 | 0.1 | 217 | 233 | 127 |
| Iowa | 0.0 | 0.0 | 0.0 | 0.2 | 0.0 | 0.0 | 0.0 | 0.0 | 0.0 | 0.0 | 0.0 | 0.0 | 0 | 0.1 | 73 | 80 | 183 |
| Kansas | 0.0 | 0.0 | 0.4 | 2.4 | 0.0 | 0.1 | 0.6 | 0.1 | 0.1 | 0.1 | 0.0 | 0.0 | 4 | 2.1 | 73 | 76 | 234 |
| Kentucky | 0.0 | 0.0 | 0.1 | 0.2 | 0.0 | 0.0 | 0.0 | 0.1 | 0.1 | 0.0 | 0.0 | 0.0 | 1 | 0.2 | 138 | 147 | 172 |
| Louisiana | 0.7 | 0.4 | 0.7 | 0.7 | 0.7 | 0.6 | 1.0 | 1.5 | 2.0 | 2.7 | 1.2 | 0.7 | 13 | 3.7 | 182 | 187 | 203 |
| Maine | 0.0 | 0.0 | 0.0 | 0.0 | 0.0 | 0.0 | 0.0 | 0.0 | 0.0 | 0.0 | 0.0 | 0.0 | 0 | 0.0 | 20 | 23 | 138 |
| Maryland | 0.0 | 0.0 | 0.0 | 0.0 | 0.0 | 0.0 | 0.0 | 0.0 | 0.0 | 0.0 | 0.0 | 0.0 | 0 | 0.0 | 73 | 77 | 38 |
| Massachusetts | 0.0 | 0.0 | 0.0 | 0.0 | 0.0 | 0.0 | 0.0 | 0.0 | 0.0 | 0.0 | 0.0 | 0.0 | 0 | 0.0 | 83 | 84 | 37 |
| Michigan | 0.0 | 0.0 | 0.0 | 0.1 | 0.0 | 0.1 | 0.1 | 0.1 | 0.0 | 0.0 | 0.0 | 0.0 | 0 | 0.1 | 188 | 189 | 253 |
| Minnesota | 0.0 | 0.0 | 0.0 | 0.4 | 0.3 | 0.0 | 0.2 | 0.0 | 0.2 | 0.1 | 0.0 | 0.0 | 1 | 0.5 | 91 | 99 | 319 |
| Mississippi | 0.3 | 0.3 | 1.0 | 0.5 | 0.3 | 0.4 | 0.3 | 0.6 | 1.4 | 1.6 | 0.6 | 0.4 | 8 | 3.0 | 55 | 63 | 202 |
| Missouri | 0.0 | 0.1 | 0.4 | 0.7 | 0.0 | 0.1 | 0.0 | 0.1 | 0.2 | 0.1 | 0.1 | 0.0 | 2 | 0.5 | 118 | 131 | 253 |
| Montana | 0.0 | 0.0 | 0.1 | 0.3 | 0.3 | 0.1 | 2.3 | 5.1 | 3.1 | 0.7 | 0.5 | 0.0 | 13 | 14.4 | 30 | 32 | 356 |
| Nebraska | 0.0 | 0.0 | 0.1 | 0.2 | 0.0 | 0.0 | 0.3 | 0.0 | 0.0 | 0.0 | 0.0 | 0.0 | 1 | 0.6 | 39 | 42 | 223 |
| Nevada | 0.0 | 0.0 | 0.0 | 0.0 | 0.0 | 0.4 | 0.7 | 0.3 | 0.2 | 0.0 | 0.0 | 0.0 | 2 | 1.6 | 38 | 44 | 128 |
| New Hampshire | 0.0 | 0.0 | 0.0 | 0.0 | 0.0 | 0.0 | 0.0 | 0.0 | 0.0 | 0.0 | 0.0 | 0.0 | 0 | 0.0 | 16 | 18 | 43 |
| New Jersey | 0.0 | 0.0 | 0.0 | 0.0 | 0.0 | 0.0 | 0.0 | 0.0 | 0.0 | 0.0 | 0.0 | 0.0 | 0 | 0.1 | 122 | 123 | 31 |
| New Mexico | 0.0 | 0.0 | 0.1 | 0.3 | 0.5 | 1.9 | 0.9 | 0.1 | 0.2 | 0.1 | 0.2 | 0.1 | 4 | 1.4 | 54 | 58 | 233 |
| New York | 0.0 | 0.0 | 0.0 | 0.0 | 0.0 | 0.0 | 0.0 | 0.0 | 0.0 | 0.0 | 0.0 | 0.0 | 0 | 0.1 | 205 | 210 | 213 |
| North Carolina | 0.2 | 0.2 | 0.4 | 0.5 | 0.3 | 0.4 | 0.3 | 0.3 | 0.4 | 0.3 | 0.2 | 0.1 | 3 | 0.5 | 133 | 146 | 237 |
| North Dakota | 0.0 | 0.0 | 0.0 | 0.2 | 0.3 | 0.0 | 0.0 | 0.0 | 0.1 | 0.2 | 0.1 | 0.0 | 1 | 0.3 | 48 | 51 | 190 |
| Ohio | 0.0 | 0.0 | 0.0 | 0.1 | 0.1 | 0.1 | 0.2 | 0.2 | 0.2 | 0.1 | 0.0 | 0.0 | 1 | 0.3 | 256 | 261 | 159 |
| Oklahoma | 0.2 | 0.2 | 1.7 | 1.6 | 0.1 | 0.2 | 0.3 | 0.3 | 0.3 | 0.3 | 0.2 | 0.2 | 6 | 2.1 | 97 | 102 | 228 |
| Oregon | 0.0 | 0.2 | 0.3 | 0.4 | 0.5 | 0.4 | 5.6 | 5.2 | 1.4 | 1.6 | 1.9 | 0.2 | 18 | 15.5 | 38 | 41 | 396 |
| Pennsylvania | 0.0 | 0.0 | 0.0 | 0.0 | 0.1 | 0.1 | 0.1 | 0.1 | 0.0 | 0.0 | 0.0 | 0.0 | 0 | 0.1 | 267 | 269 | 208 |
| Rhode Island | 0.0 | 0.0 | 0.0 | 0.0 | 0.0 | 0.0 | 0.0 | 0.0 | 0.0 | 0.0 | 0.0 | 0.0 | 0 | 0.0 | 12 | 12 | 6 |
| South Carolina | 0.3 | 0.3 | 1.2 | 0.6 | 0.3 | 0.2 | 0.3 | 0.2 | 0.3 | 0.2 | 0.1 | 0.1 | 4 | 1.6 | 69 | 78 | 148 |
| South Dakota | 0.0 | 0.0 | 0.0 | 0.1 | 0.0 | 0.1 | 0.2 | 0.2 | 0.0 | 0.1 | 0.0 | 0.0 | 1 | 0.5 | 13 | 14 | 211 |
| Tennessee | 0.0 | 0.0 | 0.3 | 0.2 | 0.0 | 0.1 | 0.1 | 0.2 | 0.2 | 0.1 | 0.0 | 0.0 | 1 | 0.5 | 114 | 122 | 188 |
| Texas | 0.6 | 0.8 | 1.5 | 1.1 | 0.7 | 0.6 | 0.9 | 1.7 | 1.6 | 1.5 | 0.9 | 0.6 | 12 | 3.6 | 604 | 658 | 809 |
| Utah | 0.0 | 0.0 | 0.0 | 0.1 | 0.1 | 0.9 | 1.1 | 0.3 | 0.2 | 0.2 | 0.0 | 0.0 | 3 | 1.6 | 59 | 63 | 111 |
| Vermont | 0.0 | 0.0 | 0.0 | 0.0 | 0.0 | 0.0 | 0.0 | 0.0 | 0.0 | 0.0 | 0.0 | 0.0 | 0 | 0.0 | 6 | 6 | 43 |
| Virginia | 0.0 | 0.0 | 0.1 | 0.2 | 0.1 | 0.1 | 0.2 | 0.3 | 0.3 | 0.3 | 0.1 | 0.0 | 2 | 0.4 | 108 | 121 | 185 |
| Washington | 0.0 | 0.0 | 0.2 | 0.2 | 0.3 | 0.1 | 3.1 | 4.5 | 2.8 | 1.3 | 0.7 | 0.0 | 13 | 11.8 | 78 | 81 | 306 |
| West Virginia | 0.0 | 0.0 | 0.0 | 0.1 | 0.0 | 0.0 | 0.1 | 0.1 | 0.1 | 0.0 | 0.0 | 0.0 | 1 | 0.1 | 107 | 113 | 134 |
| Wisconsin | 0.0 | 0.0 | 0.0 | 0.2 | 0.1 | 0.0 | 0.0 | 0.0 | 0.0 | 0.0 | 0.0 | 0.0 | 0 | 0.1 | 99 | 107 | 223 |
| Wyoming | 0.0 | 0.0 | 0.0 | 0.1 | 0.0 | 0.2 | 0.7 | 1.0 | 0.2 | 0.1 | 0.0 | 0.0 | 2 | 1.6 | 59 | 62 | 165 |
| TOTAL | 6 | 6 | 17 | 18 | 10 | 31 | 53 | 75 | 33 | 26 | 12 | 5 | 293 | 56 | 5,378 | 5,738 |  |
| TOTAL (LOWER48) | **6** | **6** | **17** | **18** | **9** | **16** | **29** | **36** | **32** | **26** | **12** | **5** | **213** | **50** | **5,338** | **5,695** | **9,639** |
